# Polymeric Membranes

**DOI:** 10.3390/membranes11040294

**Published:** 2021-04-19

**Authors:** Vicente Compañ

**Affiliations:** Departamento de Termodinámica Aplicada, Universitat Politécnica de Valencia, C/Camino de Vera s/n, 46022 Valencia, Spain; vicommo@ter.upv.es

This Special Issue of Polymeric Membranes is dedicated to gathering research carried out within the field of polymeric membranes in different fields of application. The papers published in this Special Issue can be seen as a response to the main needs and challenges of researchers and technologists interested in all aspects of science and technology related to their application to the syntheses of polymers to obtain membranes with excellent dielectric, mechanical, chemical, electrochemical, and transport properties for application in the construction of energy devices. For this, the use of the electrospinning techniques, ionic liquids (ILs), molecular organic frameworks (MOFs), zeolitic imidazolate frameworks (ZIFs), and the preparation of mixed matrix membranes (MMMs), which combine an organic polymeric matrix with an inorganic filler, have all blossomed over the last decade. The use of fillers in polymeric matrices has been decisive in obtaining advanced composite membranes with improved transport of ions and gases through membranes, hydrogels, and fuel cell performance, essentially due to the enhancement of ionic transport and gas separation. In all of them, an attempt was made to share a common vision aimed at the search for sustainable energy sources with a marked reduction in environmental pollution so that the final product is sustainable and efficient.

The papers compiled in this Special Issue can be read as a response to the current needs and challenges in membrane development for efficient facilitated transport, in particular, for CO_2_/CH_4_ separation from oilfields associated with gas containing carbon dioxide and hydrocarbons. In this sense, Chunwei Zhang et al. [1] studied the influence of feed gas humidity, temperature, composition, and pressure on membrane separation performance to obtain the lowest cost below operating conditions. On the other hand, papers where multi-nanolayer films were investigated with the purpose of quantifying structural and barrier property materials were also included. Such materials have shown the formation of an oriented crystalline structure for well-chosen semi-crystalline polymers due to the confinement effect when the layer thickness decreases. For example, multi-nanolayer films of polyethylene (PE) and Polyamide (PA6) (PE/PA6), alternating PE and PA6 layers were investigated with the aim of understanding the morphological, structural, and barrier properties resulting in a nano-stratified structure [2]. Such structures demonstrated to be a relevant polymer pair for the industrial production of barrier films, as the crystalline morphology correlated with the barrier properties of the films in respect to different gases such as nitrogen (N_2_), oxygen (O_2_), carbon dioxide, and water. The influence of interphases on the barrier properties were discussed in light of the serial model of diffusion resistance. The novelty of this work is in the design of new membranes made up of alternating hydrophobic and hydrophilic nanolayers a few nanometers thick. For these non-compatible polymers, PE and PA6, one hydrophobic the other hydrophilic, the use of polyethylene-*g*-maleic anhydride (PEgMA) as a compatibilizer is essential in order to ensure adhesion layers between phases and, in consequence, to keep the additional properties of both polymers. This is crucial in multinanolayered structures possessing thousands of interfaces. The use of the forced assembly coextrusion process with layer multiplying elements (LMEs) allowed the creation of multi-nanolayer PE/PA6 films well-structured with continuous layers down to ~50 nm with great performance on barrier properties where the gas permeability decreases some orders of magnitude. The third paper deals with the effect of high ammonium salt concentration and temperature on the structure, morphology, and ionic conductivity of protons in solid polymer electrolyte-based polyvinyl alcohol (PVA) prepared with a high salt concentration of ammonium nitrate (NH_4_NO_3_) via the solvent casting technique. The solid polymer electrolytes (SPEs)-based PVA are aimed to be applied with electrochemical devices, such as high-powered solid-state rechargeable batteries, supercapacitors, chemical sensors, electrochromic displays, and fuel cells, due to the fact of their advantages over gel and liquid electrolytes [3]. In this work, a series of proton-conducting SPEs was prepared from PVA as the host matrix, and low-cost ammonium nitrate (NH_4_NO_3_) was used as a proton source for improving the ionic conductivity of the composite. The structural, electrical, and transport properties of the prepared samples were studied as a function of salt concentrations, frequency, and temperature to expect the possible applications of the system. The substitution of ammonium nitrate in place of lithium salts has the advantage of lower cost in comparison with existing salts currently on the market based on lithium salts.

The SPEs have gained more attention due to the fact of their extensive applications in electrochemical sensors, reactors, and proton-exchange membrane fuel cells. Research carried out recently reveals that incorporating ammonium salts into polar polymer produces proton-conducting electrolytes due to the one loosely bound of the four protons attached to nitrogen. The conclusions obtained in this work show that electrical conductivities increase from an order of 10^−9^ S/cm to 10^−5^ S/cm at room temperature and 10^−4^ S/cm at 353 K. The highest ionic conductivity at ambient temperature was achieved for PVA loaded 30 wt.% NH_4_NO_3_, which is associated with a decrease in free ions and the lowest degree of crystallinity. This observation indicates that polymer segmental motions play a crucial role in the ion transport processes in the present SPE system.

The design and realization of multiscale porous structures has been a long-standing challenge in membrane science. It is method for block copolymers (BCPs) with their self-assembly enabled nanodomains to gain the potential to make structural breakthroughs. In Reference [4], an amphipathic Janus membrane, with a hierarchical multiscale hyperporous structure, constituted by polystyrene-b-poly(4-vinylpyridine) (PS4VP) and polyvinylidene fluoride (PVDF) blocks, was designed and synthesized to provide a general, facile, and unique example for the design and synthesis of a hierarchical multiscale hyperporous membrane for interfacial catalysis. For this, hydrophobic PVDF dominated one side of the membrane, providing continuous macroporosity of approximately 1 μm in pore size, providing space for the formation of finer hierarchical structures, while hydrophilic PS4VP with nanopores that formed inside the macroporous channels of PVDF via a self-assembly approach dominated the other side. The self-assembly of PS4VP into well-ordered pores with high porosity and low tortuosity, enhanced the transport of both substrates and products through the membrane at the interface. Later, Candida rugosa lipase (CRL), as a model biocatalyst, was immobilized in the PS4VP nanopores via injection. The immobilized lipase was exactly suspended at the interface of the organic and aqueous phases, owing to the amphipathic property of the Janus membrane. With the composite membrane obtained, the authors observed a great increase in the specific activity than the free lipase, even after the same cycles of reuse, giving good stability to the membrane in respect to the interfacial catalysis. Therefore, Janus membranes with nanoporosity and immobilized lipase with a high catalysis efficiency and operation stability obtained via a simple method allow us to expect that this kind of membrane is useful in applications such as catalysis, biosensors, and biomedical devices and also provide an effective idea for the design and realization of nano-multiscale porous structures.

Hadi et al [5] studied the influence of a glycerol plasticizer on the ionic conductivity of the chitosan (CS)-based nanocomposite polymer electrolytes (NCPEs). For this, polymer CS with higher molecular weights, which was obtained from deacetylated chitin, silver nitrate (AgNO_3_) salt dopant, aluminum oxide (Al_2_O_3_) nanoparticle, acetic acid (CH_3_COOH), and glycerol (C_3_H_8_O_3_) plasticizer, were used as raw materials to prepare plasticized NCSPE films. For this, the authors in this work used a constant weight percent ratio of 40 wt.% AgNO_3_ salt and 3 wt.% Al_2_O_3_ nano-filler to fabricate CS-based NCSPE film, and then the glycerol plasticizer was used in different concentrations to enhance the conductivity. In this study, several electric and electrochemical investigations were performed showing that the plasticized CS-based NCPEs can be used for EDLC device applications. Additionally, the electrolyte with the greatest value of conductivity was employed as an electrode separator in the construction of the EDLC devices. The specific capacitance value for the best conducting plasticized CS-based NCSPE system was found to be scan rate dependent, and it was 43.7, 44.01, and 47.78 F/g at the scan rates of 50, 20, and 10 mV s^−1^, respectively. The transport number found was approximately 0.985. 

Different strategies and approaches to producing porous membranes with high permeability, good rejection rates, and antifouling properties have been conducted by many researchers for water purification. Molka Nafti Mateur et al. [6] focused their study on porous gelatin membranes obtained from Pickering emulsions stabilized with hexagonal boron nitride nanosheets (h-BNNS) with the purpose of stabilizing micro-droplets of castor oil in a continuous homogeneous gelatin solution to be applied as a polyelectrolyte to enhance ultrafiltration. The steps in the membrane’s preparation were observed to be strongly influenced by the porous structure. Specifically, the duration of the drying time after emulsion casting and the duration of the cross-linking step affected the membrane’s pore size, hydrophobicity, water swelling, and water permeability. By controlling these two steps, membranes could be designed with pore sizes between 0.39 and 1.60 μm and display pure water permeability between 150 and 506 L h^−1^ m^−2^ bar^−1^. The results highlight the possibility of tailoring membrane porosity by means of the rigorous control of the elaboration process parameters. This method is sustainable since it uses mainly water as a solvent.

The work of Ahmad S. F. M. Asnawi, et al. [7] proposes the use of metal complexes as a novel approach to enhance the amorphous phase and improve the electrochemical double-layer capacitor (EDLC) performance of plasticized proton-conducting CS-based polymer electrolytes. In this manuscript, the glycerolized CS–NH_4_F polymer electrolytes incorporated with zinc metal complexes were crucial for EDLC application. The ionic conductivity of the plasticized system was improved around two orders of magnitude with the addition of Zn for the metal complex. In a previous work by the same authors, it was shown that metal complexes are crucial to improving the amorphous phase of polar polymers [5]. The increase in the amorphous phase is promising to increase direct current ionic conductivity. Thus, the main objective of increasing the zinc metal complex into the CS-based electrolyte is to improve an amorphous phase for ion conduction. The highest conducting plasticized system among the electrolytes with and without the metal complex was employed for EDLC fabrication. The results indicated that the metal complex can enhance the performance of the fabricated EDLC device. The XRD and impedance investigations explored more evidence. In this study, the charge–discharge profile was obtained for the system incorporated with the metal complex. The obtained specific capacitance was compress between 69.7 and 77.6 F/g. The energy and power densities became stable from 7.8 to 8.5 Wh/kg and 1041.7 to 248.2 W/kg, respectively, as the EDLC finalized the cycles. From such results, is allow us to assume that EDLC is more preferable than other supercapacitors because it has high power density, high durability, and better thermal stability. On the other hand, biopolymer SPEs can reduce plastic waste pollution since they are made of natural resources and are highly biodegradable.

The research and critical discussion in these seven contributions contained in this Special Issue evidence the roles and importance that composite membranes have in the field of correlation between the chemical nature of polymer matrix, its structure, and preparation with the electro-transport properties, selectivity, and performance in their applications. I can assure you that the content exposed in these works will serve to stimulate growth and progress in each of the areas of interest. I thank all our friends and colleagues who contributed papers to develop important aspects on this topic addressed in this Special Issue.
